# Relevance and Premises of Values-Based Practice for Decision Making in Brain Health

**DOI:** 10.3390/brainsci14070718

**Published:** 2024-07-17

**Authors:** Panagiotis Alexopoulos, Iracema Leroi, Irina Kinchin, Alison J. Canty, Jayashree Dasgupta, Joyla A. Furlano, Aline Nogueira Haas

**Affiliations:** 1 Mental Health Services, Patras University Hospital, Faculty of Medicine, School of Health Sciences, University of Patras, 26504 Patras, Greece; 2 Global Brain Health Institute, Trinity College Dublin, D02 X9W9 Dublin, Ireland; iracema.leroi@gbhi.org (I.L.); kinchini@tcd.ie (I.K.); alison.canty@gbhi.org (A.J.C.); jayashree.dasgupta@gbhi.org (J.D.); joyla.furlano@gbhi.org (J.A.F.); aline.haas@gbhi.org (A.N.H.); 3 Department of Psychiatry and Psychotherapy, Klinikum Rechts der Isar, Faculty of Medicine, Technical University of Munich, 81675 Munich, Germany; 4 Patras Dementia Day Care Centre, 26226 Patras, Greece; 5 Centre for Health Policy and Management, Trinity College Dublin, D02 PN40 Dublin, Ireland; 6 Wicking Dementia Research and Education Centre, University of Tasmania, Hobart, TAS 7001, Australia; 7 Department of Healthcare Management, Chitkara University Punjab, Chandigarh-Patiala National Highway, Rajpura 140401, Punjab, India; 8 Samvedna Care, Samaspur, Gurgaon 122002, Haryana, India; 9 Faculty of Health Sciences, McMaster University, Hamilton, ON L8S 4L8, Canada; 10 School of Physical Education, Physiotherapy and Dance, Universidade Federal do Rio Grande do Sul, Porto Alegre 90035-190, Brazil

**Keywords:** value diversity, brain health, delivery of healthcare, ethics, evidence-based medicine, meta-values

## Abstract

Brain health is a complex concept, shaped by a plethora of determinants related to physical health, healthy environments, safety and security, learning and social connection, as well as access to quality healthcare services. Decision-making in this complex field is characterized by diverse values, potentially conflicting interests, and asymmetrically influential stakeholders. Values-based practice (VBP) is a toolkit for balancing values in a democratic and inclusive way, so that every stakeholder feels a sense of ownership over the decision made. In VBP, the emphasis is on good process rather than on pre-determined ‘correct’ outcomes. Based on two case vignettes, we highlight the relevance of the ten principles of VBP for balancing different values to the satisfaction of those directly concerned, in a given decision-making process. In addition, we argue that the successful implementation of VBP in the complex area of brain health, as well as in other fields, is premised on higher order values (meta-values), beyond mutual respect and the legal, regulatory, and bioethical framework. These include mutual regard, reciprocity, autonomy, and an egalitarian attitude towards VBP procedures and involved stakeholders.

## 1. Introduction

Brain health, an emerging overarching concept, shapes a complex field of health-related values. The term “values” is understood here in a wide manner. It refers to interests, pleasures, viewpoints, likes, preferences, duties, moral obligations desires, wants, goals, needs, aversions, and attractions, and it is not restricted to the shared values of legal, regulatory, and bioethical frameworks [1]. It expresses what matters or what is important to people. Brain health encompasses the cognitive, sensory, social-emotional, behavioral, and motor aspects of brain functioning, enabling individuals to achieve their potential for both health and wellbeing over their life course, independent of the presence or absence of disease [2,3]. It depends on a continuous, complex interplay between a plethora of interconnected determinants pertaining to physical health, healthy environments, safety and security, learning and social connection, and access to quality services (Table 1) [3].

Brain health is heavily influenced by determinants far beyond the control of individuals and their families, which operate at a population level [3]. The breadth of determinants of brain health makes it a concept that is informed and supported by a range of stakeholders with highly diverse values, and hence potentially conflicting interests and potential power imbalances. Protection from radiation or infections, for instance, or equitable access to essential medicines and health services, is contingent on interacting social, financial, and political factors that can often be only minimally influenced by individual or small group initiatives. Even more, they are scarcely impacted by individuals with impaired capacity to advocate for themselves (e.g., the very young, the very old, the sick, and those who live in poverty), particularly when the willingness of societies and governments to act on behalf of their citizens is low [4,5,6]. Τhus, it is very likely that the values of those directly concerned may not be sufficiently influential or informed, although the decision-making process crucially affects their own brain health and quality of life. Thus, a framework that fosters equity and balance in values in the complex area of brain health-related decision-making is needed.

Here, we outline the relevance of a new approach to working with values in healthcare, called values-based practice (VBP) for decision-making in brain health. VBP improves the visibility of different values being at play in a given decision-making context and contributes to clarifying the interactions between them [7]. To our knowledge, VBP has not been previously proposed and discussed as a useful tool for decision-making in the context of brain health. We use two case vignettes related to the challenges associated with climate change in different parts of the world to illustrate our points. In addition, we discuss possible obstacles to VBP implementation in decision-making in brain health and values of a higher order (meta-values), which may facilitate its application.

## 2. Values-Based Practice

VBP offers a process for working with complex and conflicting values in health-related decision-making [8]. In VBP, the term ‘values’ is conceptualized in a relatively wide manner, encompassing interests, pleasures, likes, preferences, duties, moral obligations desires, wants, goals, needs, aversions, and attractions, as well as other kinds of selective orientations. People’s values express what matters or what is important to them. The introduction of VBP into different fields of healthcare (e.g., occupational therapy, orthopedics, primary care, psychiatry, psychology, community mental health practice, child psychiatry, educational psychology, radiotherapy) has been proposed, and training materials for healthcare professionals have been developed [8,9,10,11]. The inclusiveness of VBP regarding value diversity and its focus on the values of those directly concerned in each decision contribute to the transparency of the decision-making process and foster communication and shared decision-making, respectively. Based on the legacy of the Popperian open society [8], VBP treats values in the same way that democracy treats ideas and human voices. Hence, the VBP decision-making toolkit is neither restricted to ethical codes, nor does it prioritize one value over others. It also does not endorse certain values over others, provided that the values concerned are compatible with the mutually shared values of the relevant legal, regulatory, and bioethical frameworks. These frameworks are treated as values of a higher order (meta-values) compared to those involved in the process of the decision-making process in question.

The emphasis of VBP is on good process, rather than pre-determined ‘correct’ outcomes [12,13]. In VBP, values become subject to a process of natural selection, as the weaker, i.e., the less relevant for health promotion and combating disease, give priority to the stronger ones, under the specific circumstances of each particular case [8,14,15]. Acknowledging and accepting value differences between stakeholders results in the creation of a culture of mutual respect and responsibility and in building up a positive relationship between all those concerned, so that everyone feels a sense of ownership of the decision made [12,15]. The aim of VBP is accepting and navigating dissensus [7,16], i.e., balancing different values sometimes one way, sometimes another, based on the particular context at the time of each decision. In VBP, the perspective of health service users (i.e., individuals or community seeking to protect their brain health), their views, needs, values, competencies, resources, and aspirations are the ideal starting point for any decision, even in contexts where powerful socioeconomic and other interests may be at stake [6].

The ‘good process’ of VBP is safeguarded by ten principles [15], as shown in Table 2. Four of these principles pertain to clinical skills and practice: raising awareness regarding the involvement of values in a given decision-making process; using a clear reasoning strategy to explore value diversity; acquiring knowledge about the values and facts that may be relevant to different contexts; and having good communication skills. Two further principles underscore the importance of person-centered and multidisciplinary health service delivery. Other principles focus on the fact that all decisions are based on both values and facts. The former become noticeable particularly when they are diverse or conflicting, especially in environments where a variety of choices are at the disposal of service users. The final principle of VBP underscores the significance of partnerships in decision-making by including both service users and providers.

In the following lines, the relevance of VBP for brain health is highlighted through answering the following four questions in each one of the two case vignettes: (i) Is the brain health of the central person at risk? (ii) Who are the main stakeholders involved in the decision-making process? (iii) What are the main values at play in each case, and (iv) whether and how the VBP principles are relevant for decision-making and safeguarding the protection of brain health of people concerned in these two cases? The answers to these questions rely on consensus discussion among authors and are informed by both clinical experiences and the literature. In the second part of the paper, we try to answer the question on which meta-values, beyond the mutually agreed values, the overcoming of possible barriers to the implementation of VBP in brain health real-world settings is premised.

## 3. Case Vignettes

Changing climate conditions and environmental pollution may disturb brain physiology, facilitate the transmission of pathogens, increase the risk of brain infections, food contamination, and shortages, potentially resulting in malnutrition and poisoning, which affect brain health. In addition, climate-driven natural disasters and their socio-economic ramifications exert a permanent influence on the mental health of affected individuals [17,18]. Based on the list of determinants of brain health, the case vignettes are hypothetical and derive from authors’ clinical experiences, the literature, and reports of people with brain disorders and their care partners. They aim to illustrate the relevance of VBP and its principles for decision-making in the context of climate change threats to brain health. They do not aspire to cover the entire list of brain health determinants.

### 3.1. Case Vignette 1

The case of Janaina and her family, who are grappling with the devastating results of a rampant forest fire, is presented in Table 3.

#### 3.1.1. Impact on Brain Health

The brain health of Janaina and her family is significantly influenced by the disaster with which they are confronted. In order to maintain their current lifestyle, they would need to migrate to other sections of the forest, otherwise inevitable changes in their nutritional habits would result in poorer brain health (e.g., lower consumption of vegetables). Moreover, such catastrophes lead to degradation of the quality of air and water, while social networks are at risk, since the consequences of an uncontrolled forest fire may trigger the migration of other members of the community in different directions in order to safeguard their survival. Moreover, the brain health of Janaina and her family members is negatively influenced by the dearth of culturally relevant services to support them, as well as by the physical insecurity resulting from the uncontrolled deforestation of their ancestral land. In addition, there is an increased risk of mental health problems, which may manifest in conditions such as posttraumatic stress disorder, adjustment disorder or depression. Natural disasters can lead to both short- and long-term psychological distress and impose a significant burden of mental health conditions on individuals and the community affected by them [19,20]. Such mental disorders negatively affect brain health and embody risk factors for subsequent cognitive decline [21,22].

#### 3.1.2. Stakeholders in the Decision-Making Process

In the decision-making process regarding staying on their ancestral land or migrating, many stakeholders are involved. Janaina and her family are central to this decision. Furthermore, relatives and close friends of the family influence the decision too, since they are the significant others who shape the community which Janaina is so reluctant to abandon. In addition, the local community leaders, who are, commonly, deeply aware of the needs, wishes and dynamics of the community within the frames of its traditions and cultural background, as well as the regional and federal authorities, being in charge of the management of the emergency situation and its short- and long-term ramifications, also influence the process. Moreover, medical and non-medical healthcare professionals and scientists (e.g., biologists, geologists) also contribute to developing strategies to manage the variable detrimental effects of the disaster at local and regional levels. Last but not least, private construction corporations, non-governmental organizations being active in promoting the protection of human rights and traditions of Indigenous people and/or refugees and environmental protection activists may be among those impacting the decision-making process.

#### 3.1.3. Values Involved in Decision-Making

Various values are in play in the decision-making for Janaina (Table 4). Her ambivalent stance is shaped by values related to health protection, security, the survival of her family, dedication to cultural identity and the lifestyle of the community in which she has grown up and to which she belongs. The significant others in her life may be motivated by their aim to survive and protect their life and health, while putting less emphasis on adhering to their traditional lifestyle. Moreover, community leaders in most cases seek a pragmatic compromise between life and health protection and the maintenance of the traditional ways of life, community ties, and cultural heritage, which under such circumstances may be an enigma. Furthermore, the stances of the regional and federal authorities in the decision-making process are affected by the humanitarian and environmental crisis impact, public pressure, available services to manage the emergency situation, available financial resources, as well as the cultural sensitivity and corruption temptations of the politicians and civil servants involved in decision-making. Healthcare professionals and scientists strive to protect human life, health, and the wealth of natural resources, respectively, even though they may approach the difficulties of Janaina and her family differently, through the lens of their professional identity. Private corporations are mainly driven by their financial interests in the decision-making process but also, ideally, by their engagement in corporate social responsibility policies. On the other side, non-governmental organizations’ involvement is mainly driven by the protection of human rights and of the cultural identity of Indigenous communities. Environmental protection activism paves the way towards addressing pollution, climate change and natural resource depletion and takes the initiative to drive change. All these different aims, interests, needs, preferences, competencies, resources, aspirations, beliefs, principles, and agendas form an explosive admixture which may paralyze or even undermine efforts to protect the brain health of Janaina and her family, which is at risk because of the consequences of the rampant forest fire.

#### 3.1.4. Relevance of VBP for Decision-Making in the Case of Janaina

In the case of Janaina, VBP may offer a pragmatic platform for reaching a decision that is balanced and inclusive of different values. Firstly, in VBP, the perspective of the people directly concerned is prioritized over the values of other stakeholders (the “patient perspective” principle). In such a way, the perspective of Janaina will neither be overshadowed by the values of more powerful actors of the decision-making procedure nor will it be homogenously treated as identical to that of other members of the Indigenous community. In combination with facts (the “two feet” principle), value diversity is the solid basis for accepting and navigating dissensus, i.e., balancing legitimately different values sometimes one way, sometimes the other way (the “multi-perspective” principle). Decisions are amenable and responsive to contextual changes. For instance, a few months after the forest fire, as media and public attention wane, Janaina’s perspective of leaving the fractured and probably morally injured community could shift. She might consider moving to a small settlement established by regional authorities for disaster victims. This relocation could offer her family secure livelihood and improved access to permanent healthcare and social services, urgently needed due to the catastrophe’s aftermath (e.g., the post-traumatic stress disorder of her husband). In addition, the impact of influential actors on decision-making (e.g., private corporations’ interests, local, regional, and national authorities) is counterbalanced by the legal and regulatory frameworks, mutual respect, and the bioethical values of autonomy, justice, beneficence, and nonmaleficence. Additionally, the toolkit’s emphasis on good process highlights the importance of clear reasoning process over outcome (the “space of values” principle).

VBP allows space for subjective information derived from personal experience and openness to improving knowledge, acquired through empirical and philosophical methods, about the values of others involved in decision-making (the “values myopia” principle). The values, principles, competencies, resources, and the needs of Janaina and her family should not only be articulated and heard, but understood in the cultural context of the Indigenous community, so that the decisions that are made are culturally sensitive. As evidenced by this case, the principles of VBP are important for brain health optimization in low- and middle-income countries, as well as in remote and rural communities of high-income countries.

A dilemma which may arise in non-purely clinical cases regards the stakeholder who plays the role of the moderator of the decision-making process in VBP. Even in such cases, medical or non-medical healthcare professionals can pragmatically moderate the process, since they are the stakeholders who are more deeply aware of the principles of VBP and have obtained the necessary training in its implementation. In addition, based on the legal and bioethical foundations of clinical practice, they serve as advocates for the needs, wishes, preferences, and further values of the people who are confronted with brain health-related challenges, ensuring that the process abides by the “patient-perspective” principle irrespective of power imbalances.

### 3.2. Case Vignette 2

The case of David, who is hospitalized because of delirium, is presented in Table 5.

#### 3.2.1. Impact on Brain Health

Delirium is a very common neuropsychiatric syndrome related to brain dysfunction [23]. It is characterized by an acute onset of fluctuating impairment in consciousness and cognition, particularly attention, which may be coupled with psychotic symptoms and mood disturbances. Neuroinflammation, cerebrovascular dysfunction, changes in brain metabolism, neurotransmitter imbalance and impaired neuronal network connectivity are implicated in the development of delirium. Of note, delirium is a risk factor for subsequent cognitive decline leading to dementia, while people with cognitive impairment due to a neurodegenerative disease such as Parkinson’s disease are at high risk for developing delirium. Thus, delirium clearly signifies that David is confronted with challenges related to his brain health.

#### 3.2.2. Stakeholders of the Decision-Making Process

In the decision-making process regarding the management of delirium, which is a very common syndrome of brain dysfunction in older adults and pertains to a high risk of developing dementia and mortality [23,24], several stakeholders are involved. In addition to David, his children, and medical and non-medical hospital healthcare staff directly participate in the management of his symptoms during his hospitalization. Furthermore, the head of the ward nursing team and the hospital head pharmacist who oversee the human resources available during the different shifts and the medicines available at the hospital, respectively, influence the decision-making process, too. Since the type and extent of available health resources, i.e., financial resources (health spending) and human resources of the hospital (e.g., adequate nursing and occupational therapy staffing, patients’ room interior design) are contingent upon the decisions of the hospital leadership, including the hospital chief executive officer (CEO) and the board of directors, this indirectly influences clinical decisions and the management of David’s delirium symptoms.

#### 3.2.3. Values Involved in Decision-Making

Different values contribute to the decision-making process regarding the way the difficulties of David will be managed (Table 6). The protection of David’s health is a concern of all stakeholders, but not the main concern for all of them. For instance, in addition to protecting and restoring his health to enable him to live independently again, David may wish to minimize the burden that his needs for support and care pose on his children. Unfortunately, time periods of lucidity during which David can clearly express his wishes are a few and limited [25]. His children are confronted with a situation that necessitates their presence at the hospital. Even though the eldest son prioritizes his support to his father over his work and other family duties, the second son emphasizes his limited availability due to other commitments.

The physician in charge of David is aware of recent scientific findings highlighting that the therapeutic effects of antipsychotics in delirium are possibly restricted to sedation, without impact on the duration of the symptoms [26], while treatment with more cost-effective, older options will almost certainly lead to an acute exacerbation of his parkinsonian symptoms [27]. On the other hand, the management of David’s symptoms with non-pharmacological interventions (e.g., withdrawing precipitating or distressing factors, provision of sensory, emotional and environmental support) [23] makes the almost permanent presence of ward staff and/or family members next to his bed inevitable. The head of the ward nursing staff aims to reach a reasonable compromise between providing patients with high standard nursing care and simultaneously protecting the rights and health of the ward staff (e.g., safeguarding their entitled time-off). The hospital head pharmacist seeks a pragmatic balance between the increased costs of medications for an individual and the need to consider all other patients’ needs in the hospital. The leadership of the hospital works on the financial sustainability of the hospital, i.e., having control over costs and adhering to annual financial planning, as well as on providing high-quality services.

#### 3.2.4. Relevance of VBP for Decision-Making in the Case of David

The decision-making process regarding David’s treatment can benefit from VBP. Due to consciousness disturbances, David may not be able to express himself adequately, but the “patient perspective” principle of VBP ensures his views are considered during moments of alertness or lucidity, prioritizing his preferences while ensuring his safety and health, guided by the bioethical principle of nonmaleficence. While VBP addresses value diversity, it does not disregard scientific evidence (the “science-driven” principle). The physician will collaborate with David and his children to make an evidence-informed and value-congruent decision, weighing the harms and benefits of each treatment option. Of note, scientific progress regarding the pharmacological and non-pharmacological interventions for delirium in people with neurodegenerative diseases has opened up choices. Values shape decisions between scientifically sound choices. If, for instance, only one therapeutic strategy was available, or if the two sons had the same availability to support their father in hospital, the role of values would have remained silent in the case of David (the “squeaky wheel” principle). Ethical reasoning in VBP explores value diversity, ensuring that each actor’s perspective is understood without imposing one value over others (the “space of values” principle). VBP allows for open discussion and reflection on different value perspectives, fostering the fruitful interaction between David, his sons, and the medical and non-medical staff. In VBP, decisions are made collaboratively, balancing different values according to the circumstances (the “who decides” principle), within the shared meta-values of legal, regulatory, and bioethical frameworks. This ensures that all those involved have a sense of ownership over the decisions made (the “how it is done” principle).

## 4. Obstacles and Facilitators of VBP in Brain Health

In the realm of VBP, dissensus is embraced, and various, potentially conflicting values are reconciled to meet the needs of all stakeholders, adapting to changing contexts. While mutual respect and locally agreed frameworks of shared values, as expressed in legal, regulatory and bioethical frameworks, are essential, they may not be sufficient for the successful implementation of VBP, especially in complex fields such as brain health. Aspects of brain health complexity that could hinder successful VBP implementation include the following:

- The breadth of brain health determinants, covering the entire life span, from conception to death, and regarding factors far beyond clinical practice such as, for instance, safety, security, learning, and social connection, as well as a plethora of stakeholders, may render decision-making in brain health a challenging task;
- The power asymmetries between stakeholders involved in decision-making, which may be extreme and subsequently undermine good process (e.g., imbalance of power between private construction corporations and healthcare professionals in the case of Janaina, or the possible indirect influence of hospital leadership on the clinical decisions related to the management of David’ delirium symptoms);
- Differences in professional and cultural backgrounds and ethos of stakeholders that may impede communication and commitment to good process such as, for example, the cultural and professional differences between the Indigenous local community leaders and the representatives of federal authorities, in the case of Janaina.

The successful implementation of VBP in brain health and other complex fields implicitly relies on several meta-values beyond mutual respect and the legal, regulatory, and bioethical frameworks.

### 4.1. Beyond Mutual Respect: Mutual Regard

A premise of VBP, as depicted by Fulford, is mutual respect between decision-makers [15]. Nonetheless, mutual respect, whether achieved by everyone maintaining a respectful distance from others and their values or reflecting a defensive mentality and stance, may be insufficient for navigating dissensus and making pragmatic decisions. For instance, in the case of Janaina, members of the local and regional authorities and/or the private construction corporations may respect Janaina’s community, but this does not suffice for the constructive engagement between stakeholders involved in the decision-making process. Something stronger than mutual respect is needed. Without openness to change, modification, and compromise, which are endorsed by the anti-dogmatism of VBP [28], the different views and values cannot be balanced in a way that, at the end of the process, all involved stakeholders feel a sense of ownership of the decision made.

Mutual regard can facilitate the implementation of VBP in decision-making related to brain health. Compared to mutual respect, mutual regard denotes greater solidarity, loyalty to the less fortunate, less reluctance to trust and compromise, and a spirit of openness to constructive cooperation [29], and it may more effectively promote mutual understanding. In such a way, it safeguards the empathic exploration of the values of all VBP parties and catalyzes the process towards accepting and navigating dissensus [28,30]. Mutual regard fosters a spirit of openness to constructive interactions [29], facilitates engagement with the diverse values of people involved in decision-making and widens value horizons instead of closing down on just mutual respect.

### 4.2. The Implicit Premise of Commitment to Good Process over Outcome: Reciprocity

The insistence of VBP on good process instead of good outcomes presupposes a deeply rooted trust in reciprocity, safeguarding trust in the decision-making process. Reciprocity is the notion that the ways values are treated within the VBP procedure must be defensible by appealing to arguments that reasonable decision-making process participants can accept in a given context [31]. Open, critical discussion and the individual’s ability to be both reasonably critical and self-critical are required for a dialogue-induced natural selection of values [32]. In the case of David, the decisions were initially made without his participation. His children acted reasonably critical and self-critical in his best interests in the process of making pressing decisions, while other decisions were put off until David could participate in the process.

A good outcome in VBP is an outcome derived from a process properly conducted [15]. The term “properly conducted” could be understood as a process underpinned by reciprocity, since reciprocity sets the stage for balancing different values in a way adjusted to the particular circumstances of each time point and context. Reciprocal justification is a substantive meta-value within VBP, as it serves as a standard for assessing and justifying the legitimacy of VBP outcomes and fosters trust in the decision-making process.

### 4.3. Procedural and Substantive Dimensions of Autonomy in VBP

Autonomy is another meta-value on which VBP is premised in the context of brain health and other complex settings of decision-making [33,34]. In VBP, everybody involved contributes to decision-making, reflecting a combination of different values. For instance, the physician in charge of David may have favored the most cost-effective medication despite recent scientific evidence and the recommendations of international medical societies if the hospital faced budget deficits and operated within strict austerity policies. The physician is in this case confronted with dilemmas arising from internal conflicts between values. This is not a rare constellation in the real world [35,36,37,38,39,40]. Stakeholders can present values in the process of decision-making which they would not normally prioritize if they were free to make their own choices (e.g., medication cost-effectiveness vs. therapeutic effect maximization). This is, in effect, a violation of the democratic value of autonomy and can impede on accepting and navigating dissensus because stakeholders who present values which they would normally not prioritize can scarcely have a sense of ownership of the decision made at the end of the decision-making process. Besides its procedural implications (active participation in decision-making, free expression of opinions and views), autonomy in VBP has crucial substantive dimensions [33,34]. It provides the necessary space for an individual to decide which of their own values should be prioritized, so that they contribute to shaping the outcome decision to the satisfaction of both themselves, and all others involved. Autonomy embodies, in fact, a value of higher order, a meta-value that is presupposed by a democratic procedure such as VBP.

### 4.4. Egalitarian Attitude towards VBP Procedures and Participants Despite Power Asymmetries

An egalitarian attitude of each VBP stakeholder towards decision-making process and other stakeholders facilitates the balance of different values implicated in each particular brain health decision. In decision-making related to brain health determinants, power asymmetries are invariably present. Asymmetries arise from differences in expertise, specific knowledge, skills, political and economic power, health and social status, as well as from the necessity of revealing personal and intimate information or being in danger and great need. The shadow of power asymmetry may impede the open expression of the thoughts, wishes, desires, fears, and values of the less powerful stakeholder, such as Janaina or David in the scenarios described.

Power asymmetries influence both the agenda setting (i.e., the most powerful stakeholder can predominantly set the agenda of discussions) and the process of balancing conflicting values. In open societies, the impact of the most powerful actors, threatening the democratic process, is counterbalanced by the rule of law and constraining power sharing [41]. Unfortunately, there is no guarantee that the VBP dialogue-induced natural selection of values can remain immune to the distorting influence of prevailing interests (e.g., the financial interests of the private construction corporations in the case of Janaina and her family). Nonetheless, an egalitarian attitude towards the VBP process and other stakeholders is a VBP meta-value [39,42], which can reduce the distorting influence of power asymmetries. Moral egalitarianism is here understood as a meta-level preference according to which people ought to relate as moral equals [39,40,43]. In line with moral egalitarianism, healthcare service providers and users can both be understood as being experts in clearly distinct fields in shared decision-making [44,45]. Healthcare service users are experts of their lived experience, while healthcare and other professionals are experts in their occupational fields. The egalitarian attitude towards the VBP process and participants embodies a catalyst of constructive participation in the process of accepting and navigating dissensus [46].

## 5. Concluding Remarks

Brain health is an emerging concept, the determinants of which cover the entire lifespan and are related to health, socioeconomic and epidemiological parameters and a plethora of stakeholders. VBP, as an open and flexible approach to dealing with value diversity, may contribute to the decision-making process pertaining to brain health issues to the satisfaction of all stakeholders, since it puts enormous emphasis on the importance of “good process” in overcoming the tensions that arise from value diversity. The orientation of VBP towards balancing value diversity sometimes one way, sometimes the other, based on the particular context at each point in time, cultivates positive relationships between all those concerned and leaves open space for modifications and improvements, as contexts change and adjustments become necessary.

The implementation of VBP in complex fields such as brain health necessitates openness to change and compromise, solid reasoning, flexibility and immunity to the detrimental effects of power asymmetries on good process. Thus, the successful VBP application in brain health may be facilitated by mutual regard, reciprocity, autonomy and an egalitarian attitude towards the VBP decision-making process and stakeholders. In addition to the legal, regulatory and bioethical framework, these meta-values shape the environment within which different values, regarding a particular decision to be made, are to be adequately balanced under the given circumstances and based on good process. When it comes to brain health, the principles of VBP provide an ideal framework for steering the process of democratic decision-making. Nonetheless, before final conclusions can be drawn, the development of guidelines for applying VBP in brain health decision-making, as well as conducting real-world research on the experiences of healthcare service users and other stakeholders involved in VBP decision-making regarding brain health issues, are of paramount importance for an in-depth understanding of the usefulness of VBP in brain health.

## Figures and Tables

**Table 1:** Determinants of brain health according to the position paper published by the World Health Organization [3].

Physical Health	Healthy Environments
Maternal health, intrauterine environment	Safe use of chemicals
Genetic and epigenetic factors	Protection from radiation
Nutrition	Healthy and safe workplaces and agricultural practices
Infections	Air and water quality
Neurocognitive disorders	Stable climate
Healthy behaviors	Access to preserved nature and health- supportive built environments
Traumatic injuries	
**Safety and security**	**Learning and social connection**
Physical safety	Education
Financial security	Lifelong learning
Humanitarian crises and emergencies	Nurturing care
	Social connection/social isolation
**Access to quality services**	Social networks
Integrated care at all health/social care levels	
Skilled workforce and Interdisciplinary teams	
Access to essential medicines, diagnostics, and health products	
Carer support	

**Table 2:** Principles of values-based practice (VBP) [8].

**Values-based practice is patient-centered and multi-disciplinary**
Safeguarding the person-centeredness of the decision-making process, VBP’s “first call” for information is the perspective and the values of the healthcare service user or patient group concerned in a given decision (the “patient-perspective” principle)
Fostering multidisciplinary health service delivery, VBP facilitates the fair and reasonable balancing of legitimately different perspectives of stakeholders without referring to rules prescribing “right” outcomes (the “multi-perspective” principle)
**Values-based practice depends on four key clinical skills**
Careful attention to language use is an effective strategy for raising awareness of the role and impact of values in a given context (the “values-blindness” principle)
Available empirical and philosophical methods facilitate the detection and understanding of other people’s values (the “values-myopia” principle)
The role of ethical reasoning in VBP is primarily explorative regarding value diversity, and not a pointer of “what is right”, as in quasi-legal bioethics (the “space of values” principle)
Compared to quasi-legal ethics, the role of communication skills in VBP is substantive rather than executive (the “how it is done” principle)
**Values-based practice and evidence-based practice work together**
Decision-making in healthcare stands on two feet, on values, as well as on facts (the “two feet” principle)
The role of values in healthcare-related decision-making becomes more evident when the values are diverse or even conflicting, engendering tensions and problems (the “squeaky wheel” principle)
Scientific progress opens up choices in all areas of healthcare, so that value diversity is a crucial aspect in deciding between different options (the “science driven” principle)
**Values-based practice relies on partnership between decision-makers**
In VBP, decision-making is placed back where it belongs, with service users and providers at the clinical coalface (the “who decides” principle)

**Table 3:** The case of Janaina and her family.

Janaina, a middle-aged Indigenous woman, who lives with her husband and two children in a remote area of the Amazonian Region, Brazil. Their livelihood depends on traditional land practices such as fruit picking, hunting, and fishing. Janaina and her family have been drastically impacted by a rampant forest fire, intentionally set for land clearing purposes, which spread out of control. Lacking sufficient information on the possible impacts of such fires, Janaina’s family was unprepared for this disaster. The situation is further complicated by Brazil’s social, economic, and political landscape, which has little infrastructure for emergency response in rural regions where Indigenous people often reside. Janaina’s strong cultural and social ties to the land where she is from make the idea of leaving her community unthinkable, yet there are no local, culturally relevant resources in place to assist her family during this crisis. Faced with this dire situation, she is torn between staying in her ancestral land and finding refuge elsewhere for her family’s safety. With this, mental health and quality of life concerns also arise.

**Table 4:** Examples of values and stakeholders involved in the case of Janaina and her family.

Stakeholders	Values
Janaina, family members, Indigenous community leaders, regional and national authorities, healthcare professionals	Life and health protection
Janaina, family members, Indigenous community leaders, regional and national authorities, healthcare professionals	Security and safety
Janaina, family members, Indigenous community leaders, non-governmental organizations	Protection of Indigenous cultural identity and lifestyle
Non-governmental organizations	Human rights
Janaina, family members, Indigenous community leaders	Maintenance of community ties
Civil servants, healthcare professionals, scientists	Professional values
Civils servants, politicians	Resistance to corruption temptations
Environmental protection activists, non-governmental organizations, healthcare professionals, regional and national authorities	Protection of ecosystems
Private corporations	Profit maximization
Regional and national authorities	Maintenance of social order and stability, stability-oriented national and regional fiscal policies

**Table 5:** The case of David.

David, an 83-year-old man with mild cognitive impairment due to Parkinson’s disease and chronic obstructive pulmonary disease (COPD), lives alone on the outskirts of a coastal town south of Sydney, Australia. Supported by a community nurse, he faces challenges during summer due to heat waves and bush fires. During a heat wave accompanied by a forest fire, David experienced anxiety, confusion and difficulty breathing. The community nurse treated his condition as an emergency, and David was admitted to the internal medicine ward of the local hospital, where he was diagnosed with delirium, likely due to dehydration and smoke inhalation, exacerbating his COPD. David’s adult children discussed management options with the physician and psychiatrist. Initially unable to participate in decision-making due to confusion, David’s children acted in his best interest. When lucid, the physician explained to him the treatment options, including antipsychotics, which pose a risk of worsening his movement difficulties. Other options included constant supervision by his children or non-pharmacological interventions, particularly at night, when delirium symptoms are most severe.

**Table 6:** Examples of values and stakeholders involved in the case of David.

Stakeholders	Values
David, family members, hospital medical and non-medical healthcare professionals	Health protection and promotion
David, family members, hospital medical and non-medical healthcare professionals	Independence in activities of daily living
Family members	Care partner burden minimization
Family members	Balance between care for the hospitalized family member, work and family duties
Hospital medical healthcare professionals	Minimization of the risk of side effects
Hospital medical and non-medical healthcare professionals	High-standard care
Hospital medical and non-medical healthcare professionals, medical and non-medical staff directory	Protection of healthcare professionals’ rights and health
Hospital leadership	Financial sustainability of healthcare services

## Data Availability

Not applicable.
